# Genomic contributors to atrial electroanatomical remodeling and atrial fibrillation progression: Pathway enrichment analysis of GWAS data

**DOI:** 10.1038/srep36630

**Published:** 2016-11-18

**Authors:** Daniela Husser, Laura Ueberham, Borislav Dinov, Jedrzej Kosiuk, Jelena Kornej, Gerhard Hindricks, M. Benjamin Shoemaker, Dan M. Roden, Andreas Bollmann, Petra Büttner

**Affiliations:** 1 Department of Electrophysiology, Heart Center Leipzig, Leipzig University, Germany; 2 Department of Medicine, Vanderbilt University, Nashville, TN, USA

## Abstract

In atrial fibrillation (AF), left atrial diameter (LAD) and low voltage area (LVA) are intermediate phenotypes that are associated with AF type and progression. In this study, we tested the hypothesis, that these phenotypes share common, genetically-determined pathways using pathway enrichment analysis of GWAS data. Samples from 660 patients with paroxysmal (n = 370) or persistent AF (n = 290) were genotyped for ~1,000,000 SNPs. SNPs found significantly associated with LAD, LVA or AF type were used for gene-based association tests in a systematic biological Knowledge-based mining system for Genome-wide Genetic studies (KGG). Associated genes were tested for pathway enrichment using two enrichment tools (WebGestalt and GATHER) and the databases provided by Kyoto Encyclopedia of Genes and Genomes. The calcium signaling pathway (hsa04020) was the only pathway that reached statistical significance for LAD and LVA in both enrichment tools and was also significantly associated with AF type. Within this pathway, there were 39 genes (i.e. *CACNA1C, RyR2*) that were associated with LAD, LVA and AF type. In conclusion, there is a genomic contribution to electroanatomical remodeling (LAD, LVA) and AF type via the calcium signaling pathway. Future and larger studies are necessary to replicate and apply these findings.

Atrial fibrillation (AF) is the most common cardiac arrhythmia and its natural history is characterized by an early paroxysmal course that may progress over years or decades to persistent, treatment-refractory AF. AF progression is associated with changes in atrial structure and function that are referred to as atrial electroanatomical remodeling. Evidence exists to suggest the degree of electroanatomical remodeling is linked with clinical AF phenotypes^1^. For instance, it is a common clinical observation that progression of AF from paroxysmal to persistent/permanent forms is accompanied by left atrial enlargement, atrial fibrosis (Fig. 1, top panel)^2^,^3^.

A variety of molecular pathways including myofibroblast activation, oxidative stress, inflammation or calcium handling have been implicated in different aspects and time points of the remodeling process^4^. Although genome-wide association studies (GWAS) have identified common genetic variants that increase AF susceptibility^5^ it is unknown, whether or not, clinically overlapping, remodeling-associated AF phenotypes such as left atrial enlargement, atrial fibrosis and AF type share common genetically modulated pathways.

Pathway-based analysis of GWAS data is a powerful tool to detect subtle but systematic patterns in the genome that underpin complex diseases. The approach has been successfully applied to identify novel regulatory pathways in different phenotypes, e.g. body mass index^6^, colorectal cancer^7^ or outcome of breast cancer^8^.

Here, for the first time, we use pathway enrichment analysis of GWAS data to test the hypothesis that left atrial diameter (LAD), fibrosis expressed by low voltage area (LVA) and AF type share regulatory pathways based on a polygenetic background (Fig. 1, bottom panels).

## Methods

### Patients

Six hundred-and-sixty AF patients undergoing de-novo radiofrequency AF catheter ablation between 2008 and 2013 were enrolled in the Leipzig Heart Center AF ablation registry. Demographic parameters as well as heart diseases, comorbidity, medication, LAD, left ventricular ejection fraction, AF type (persistent/paroxysmal) were evaluated.

LAD was measured in parasternal long axis view in end-systole using echocardiography. Paroxysmal AF was defined as AF episodes that self-terminated in <7days without electric or pharmacological intervention. Persistent AF was defined as arrhythmia lasting for >7 days that could only be terminated by electric or pharmacological intervention. In patients recruited between 2011 and 2013, electro-anatomical voltage mapping to characterize LVA defined as potentials below 0.5 mV was performed as previously described^9^.

The study protocol was approved by the Ethics Committee of the Leipzig University Medical Faculty. All patients signed written informed consent for study participation. All methods were performed in accordance with the relevant guidelines and regulations.

### Sample processing

Blood samples were obtained in EDTA test tubes in fasting state prior ablation. Genomic DNA was isolated using a commercial kit according to the manufacturer’s recommendations (PeqLab, Erlangen, Germany). Genotyping was performed using HumanOmniExpressExome-8-v1.2 arrays comprising about one million single nucleotide polymorphisms (SNPs) according to established protocols (Illumina, San Diego, US).

### Data analysis and statistics

#### General considerations

Typically, GWAS are performed to identify disease related SNPs whereas a p-value < 5 * 10-8 is regarded statistically genome-wide significant. This approach minimizes the number of false positives, taking into account that thousands of false negatives are excluded and biologically important information is lost. The Knowledge-based mining system for Genome-wide Genetic studies (KGG) software assigns SNPs with low significance levels (p-value < 0.05) from GWAS to genes considering the gene size and linkage disequilibrium (LD) data^10^,^11^. Significant SNPs enrichment in a gene indicates an involvement in the pathophysiology of the studied disease trait. Further verification can be achieved by testing for gene enrichment in physiological pathways as provided by the Kyoto Encyclopedia of Genes and Genomes (KEGG)^12^.

#### Analysis plan

Raw data was compiled using GenomeStudio (Illumina) software and exported to PLINK GWAs analysis package^13^. Using PLINK tool set the data was tested for consistency. Samples with a call rate <95% were excluded. Single SNPs had to meet the following criteria: minor allele frequencies (MAF) >0.01, call rate >95%, Hardy-Weinberg equilibrium (HWE) significance threshold >0.0001. Otherwise they were excluded from further analysis.

Association of genotypes with LAD was detected using linear regression with adjustment for age, gender and AF type. Association of genotypes with LVA or AF type (persistent AF) was detected using logistic regression analysis with adjustment for age; gender and AF type (only for LVA).

Illumina’s exome arrays contain specific “exm-SNPs” which were assigned to their corresponding dbSNP rs IDs prior further analysis.

The resulting SNP lists including all SNPs with a p-value less than 0.05 were used for gene enrichment. This was done with KGG^10^. R*-*square values representing linkage disequilibrium data corresponding to the CEU (Northern Europeans from Utah) population was received from 1000 Genomes project phase 1v3 to adjust for SNP dependency. SNPs were mapped onto genes according to GenCode v23 information’s. SNPs within a range of 5kb upstream and downstream of the gene were assigned to the gene. If a SNP was in the overlapping region of two genes it was assigned to both. The KGG GATES algorithm, an extension of Simes test, was used to calculate enrichment p-values incorporating functional SNP weights controlling for LD and gene length. Enrichment p-values < 0.05 were regarded statistically significant.

For pathway enrichment analysis we used the Gene Annotation Tool to Help Explain Relationships (GATHER)^14^ and WEB-based Gene SeT AnaLysis Toolkit (WebGestalt)^15^ together with the databases provided by KEGG^12^. Non-random over representation of genes from our candidate gene list in specific KEGG pathways was regarded significant when Fisher’s exact test p-value with FDR (GATHER) or hypergeometric distribution p-value corrected for multiple testing using Bonferroni correction (WebGestalt) was <0.05.

**Figure 2:**
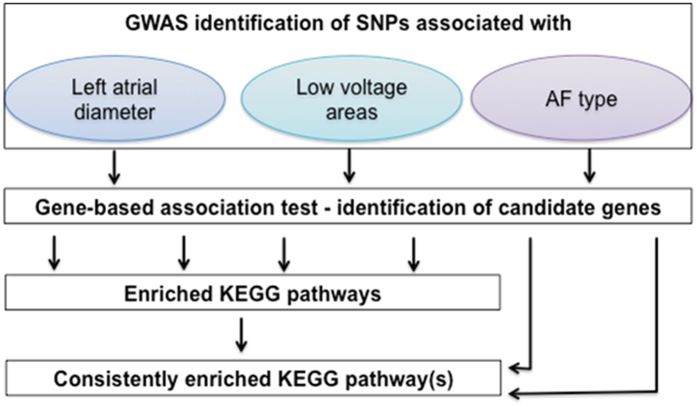
Flow chart of pathway enrichment analysis based on GWAS on LAD, LVA and AF type.

We applied a two-stage analysis plan. First, we identified consistently enriched KEGG pathways in LAD and LVA present in both enrichment tools. Second, association of those identified pathway(s) with AF type was tested with both enrichment tools (Fig. 2).

## Results

### Patient characteristics

**Figure 3:**
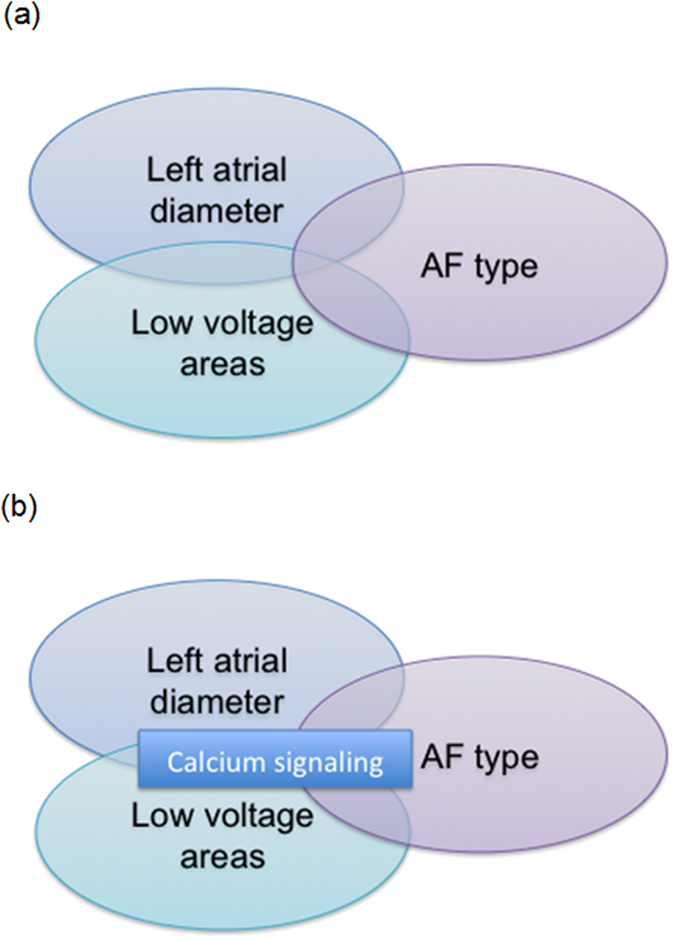
In this cohort, LAD was significantly larger in patients with LVA and in patients with persistent AF compared to patients without LVA and paroxysmal AF, respectively.LVA was more prevalent in persistent AF (**a**). Using pathway enrichment tools and KEGG databases, the calcium signaling pathway was identified to associate with LAD, LVA and AF type (**b**).

The study population included 660 unrelated patients of European Ancestry with a history of paroxysmal (n = 370) or persistent AF (n = 290, Table 1). LVA was analyzed in 163 and LAD in 538 of those data sets. LAD was significantly larger in patients with LVA (43 ± 6 vs. 46 ± 6 mm, p = 1.6E-2) and in patients with persistent AF (41 ± 5 vs. 45 ± 6 mm, p < 1.0E-3) compared to patients without LVA and paroxysmal AF, respectively. LVA was more prevalent in persistent AF (36 vs. 20%, p = 2.6E-2; Fig. 3, top panel).

Genotyping call rate in all subjects was >95% except in three samples (<85%) that were excluded from further analysis.

### Pathways associated with left atrial diameter and low voltage areas

28.062 SNPs were associated with LAD and were annotated to 10.252 genes while 24.395 SNPs were associated with LVA and were annotated to 8.918 genes. Of those, 3.425 SNPs and 1.524 genes were found in both phenotypes.

In WebGestalt, 101 KEGG pathways were associated with LAD and 61 with LVA (Supplementary Tables 1 and 2), while 55 were associated with both phenotypes. Of those, only one pathway, i.e. calcium signaling pathway (hsa04020) reached statistical significance for both phenotypes in GATHER (Table 2).

### Calcium signaling pathway and AF type

The calcium signaling pathway was significantly associated with AF type (p = 1.82E-15 in WebGestalt and p = 5.0E-3 in GATHER; Fig. 3, bottom panel). Within this pathway, there were 48 genes that were associated with LAD and LVA. Of those, 39 genes were also significantly related with AF type (Table 3).

## Discussion

### Main findings

This study is the first to explore shared common genetic pathways of clinically-overlapping, remodeling-associated AF phenotypes. We used logistic and linear regression analysis to screen a GWAS data set representing about 1 million SNPs for association with typical characteristics of AF progression namely LAD, LVA as marker of fibrosis and AF type. By applying a p-value cut off of 0.05, we identified >20,000 significant candidate SNPs per phenotype. In order to minimize false positive SNPs we used KGG software to detect non-random enrichment of SNPs in genes and furthermore annotated those genes into physiological pathways using two different pathway enrichment tools. In a two-stage association study, we first identified calcium signaling as common regulatory pathway for LAD and LVA in both enrichment tools. In a second step, this pathway was found to also associate with AF type in both tools.

Calcium signaling has been implicated as one central process of AF-associated remodeling. Moreover, mutations of genes of the cardiac calcium signal pathway may cause a number of arrhythmia syndromes. However, the contribution of the genomic background of the calcium signaling pathways to clinically-overlapping, remodeling-associated AF phenotypes is a novel and relevant finding.

### Calcium signaling in AF-associated remodeling

Several research lines have identified multidimensional roles of cellular Ca(2+) content, distribution, and handling in diverse aspects of AF initiation, maintenance and progression^16^.

Abnormal sarcoplasmic reticulum Ca(2+) leak via ryanodine receptor type 2 (RyR2) has been observed as a source of ectopic activity^17^, the hallmark of AF initiation. Abnormal calcium signaling is also implicated in atrial fibrosis, the main driver of AF maintenance and progression. Ca(2+) influx into atrial fibroblasts induces proliferation and differentiation into collagen-secreting myofibroblasts and subsequently heterogeneous conduction slowing and reentry^18^.

Angiotensin-II is an important contributor to AF-related remodeling. Inositol 1,4,5-trisphosphate receptors have been shown to mediate angiotensin-1 receptor associated Ca(2+) release^19^. Interestingly, genes encoding inositol 1,4,5-trisphosphate receptors have also been found in our study to associate with AF-remodeling phenotypes (Table 3).

In an AF mouse model, a direct causal role of RyR2-mediated sarcoplasmic reticulum Ca(2+)-leak in developing atrial structural remodeling and AF progression has been suggested. Interestingly, suppression of Ca(2+)-leak by genetic inhibition of RyR2-phosphorylation completely prevented spontaneous AF. Normalization of RyR2-mediated Ca(2+)-leak prevented atrial conduction slowing and atrial dilatation^20^. In a sheep AF model, AF progression was also associated with development of atrial dilatation and fibrosis that was, however, not dependent on Ca(2+)-leak^21^.

Despite this controversy on the *causal* role of RyR2-mediated sarcoplasmic reticulum Ca(2+)-leak for AF progression, several other studies point to important roles of the RyR2-complex in AF-related pathophysiologies such as aging, oxidative stress, heart failure and impaired glucose tolerance. For instance, Calstabin2, a component of RyR2 complex, has been identified as modulator of age-related cardiac function with augmented fibrosis, cell death and telomere length^22^. Moreover, in a mouse model, Ca(2+) leak exhibited increased atrial RyR2 oxidation, mitochondrial dysfunction, reactive oxygen species (ROS) production and AF susceptibility. Both genetic inhibition of mitochondrial ROS production and pharmacological treatment of RyR2 leakage prevented AF indicating that alterations of RyR2 and mitochondrial ROS generation form a vicious cycle in the development of AF^23^. In addition, it has been demonstrated that leaky RyR2 channels cause mitochondrial Ca(2+) overload and dysfunction in heart failure^24^. Finally, RyR2 channels play a crucial role in the regulation of insulin secretion and glucose homeostasis with leaky channels leading to impaired glucose tolerance^25^.

Interestingly, aging, heart failure^26^ and impaired glucose tolerance^27^ have been linked with AF development and progression in longitudinal epidemiological studies.

However, if and how the genotype affects the aforementioned remodeling processes in AF cohorts remains elusive, although genetic studies have identified several unique mechanisms that are discussed below.

### Contribution of calcium handling genes to AF

Variations in genes implicated in cardiac calcium signaling have been shown to cause a number of arrhythmia syndromes, including long-QT syndrome 4 (*ANK2*) and 8 (*CACNA1C*), Brugada syndrome (*CACNA1C*) and catecholaminergic polymorphic ventricular tachycardia (*RyR2*). AF is frequently present in those patients likely reflecting common mechanisms between atrial and ventricular arrhythmogenesis^28^. For instance, gain-of-function mutations in *RyR2* have been shown to predispose to catecholaminergic polymorphic ventricular tachycardia and AF by enhanced propensity for spontaneous Ca(2+) release^29^. In addition, familial and early-onset AF have been linked with rare variants of two CACNA genes with overlapping effects on the Cav1.2 (encoded by *CACNA1C*)^30^ or junctophilin 2 (*JPH2*) resulting in defective RyR2-mediated sarcoplasmatic reticulum Ca(2+) release^31^.

Interestingly, some of these calcium signaling genes have also been implicated in our study to exert effects on the remodeling process and AF progression (Table 3).

In summary, this study not only offers new insights into the genomic background of AF remodeling and progression, but it may also be viewed as hypothesis-generating, thereby paving the road for more in-depth analysis of SNPs and genes involved in calcium signaling. It is interesting to speculate that identification of genetically-controlled central pathways may eventually lead to new therapeutic targets for AF. For instance, in the experimental setting, pharmacological inhibition of RyR2 Ca(2+) leak restored atrial mitochondrial morphology and function^23^ or genetic inhibition of RyR2-phosphorylation prevented AF^20^.

### Limitations

Our study is based on small sample size and a cross-sectional study design. We addressed this by using well-defined intermediate AF phenotypes and different bioinformatics tools. Moreover, we required the genotype – phenotype correlation to be present in two pathway enrichment tools and used a two-stage approach (i.e. identification of pathways in two phenotypes and validation in a third phenotype). Single SNPs were not in the center of the study rather we focused on the most significant candidate genes from enrichment analysis. Consequently, candidate genes and pathways with lower significance levels or failing our stringent identification process could have been overlooked by this approach. Finally, detailed involvement of single pathway components in atrial remodeling and AF progression was not assessed which was beyond the scope of this study.

## Conclusions

There is a genomic contribution to electroanatomical remodeling (LAD, LVA) and AF type via the calcium signaling pathway. Future and larger studies are necessary to replicate and apply these findings.

## Additional Information

**How to cite this article**: Husser, D. *et al*. Genomic contributors to atrial electroanatomical remodeling and atrial fibrillation progression: Pathway enrichment analysis of GWAS data. *Sci. Rep.* **6**, 36630; doi: 10.1038/srep36630 (2016).

**Publisher’s note**: Springer Nature remains neutral with regard to jurisdictional claims in published maps and institutional affiliations.

## Supplementary Material

Supplementary Information

## Figures and Tables

**Figure 1:**
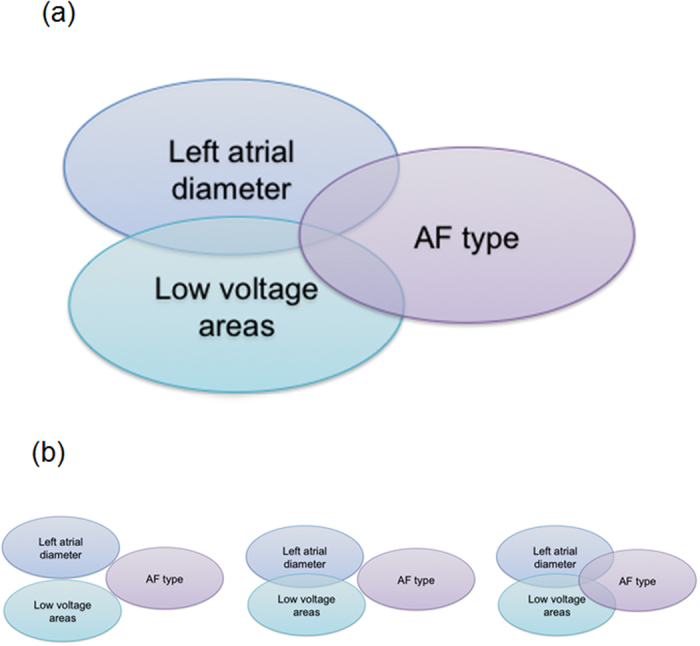
In the clinical setting, there is an overlap between left atrial enlargement and LVA that associate with AF progression (**a**) but whether or not there is a shared common genetic pathway is unknown. Three hypothetical relationships that were analyzed in this study are depicted below (**b**): LAD and LVA do not share a common genetic pathway and have no association with AF type (left); LAD and LVA do share a common genetic pathway that is, however, not associated with AF type (middle); LAD and LVA do share a common pathway that also associates with AF type (right).

**Table 1:** Patient characteristics.

	Total (n = 660)	Paroxysmal AF (n = 370)	Persistent AF (n = 290)	p value
Age (years)	60 ± 10	60 ± 10	60 ± 10	ns
Male gender (%)	68	63	75	<0.001
Idiopathic AF (%)	14	15	12	ns
Persistent AF (%)	44	0	100	na
LAD (mm)	43 ± 6	41 ± 5	45 ± 6	<0.001
LVEF (%)	59 ± 10	62 ± 7	55 ± 11	<0.001

**Table 2:** Calcium signaling pathway and its association with LAD and LVA using two enrichment tools.

Enrichment tool	Phenotype	C	O	E	R	rawP	adjP
WebGestalt	LAD	177	85	27.12	3.13	1.2E-24	2.7E-22
	LVA		70	23.5	2.98	2.4E-18	5.5E-16
GATHER	LAD	196	88			4.0E-05	5.2E-03
	LVA		73			1.2E-04	1.5E-02

C, the number of reference genes in the category; O, the number of genes in the gene set and also in the category; E, expected number in the category; R, the ratio of enrichment, rawP, the p value from hypergeometric test (Webgestalt) or Fisher’s exact test (GATHER); adjP, the p value adjusted by the multiple test adjustment. Please note that GATHER does not provide E and R.

**Table 3:** Genes of the calcium signaling pathway associated with LAD, LVA and AF type (in alphabetical order).

Gene	Full gene name	LAD	LVA	AF type
*ADCY2*	adenylate cyclase 2 (brain)	1,8E-02	4,9E-02	6,3E-03
*ADCY9*	adenylate cyclase 9	1,2E-02	4,4E-02	4,1E-02
*ADRA1A*	adrenoceptor alpha 1A	1,4E-02	1,1E-02	3,8E-02
*ATP2B2*	ATPase, Ca++ transporting, plasma membrane 2	8,3E-03	2,2E-02	4,4E-02
*CACNA1A*	calcium channel, voltage-dependent, P/Q type, alpha 1A subunit	3,0E-02	3,9E-02	4,2E-02
*CACNA1B*	calcium channel, voltage-dependent, N type, alpha 1B subunit	4,2E-02	1,8E-02	4,7E-02
*CACNA1C*	calcium channel, voltage-dependent, L type, alpha 1C subunit	7,8E-03	1,3E-02	2,2E-02
*CACNA1D*	calcium channel, voltage-dependent, L type, alpha 1D subunit	7,6E-03	1,2E-02	2,1E-02
*CACNA1E*	calcium channel, voltage-dependent, R type, alpha 1E subunit	8,7E-03	4,1E-02	3,1E-02
*CACNA1I*	calcium channel, voltage-dependent, T type, alpha 1I subunit	4,1E-02	2,4E-02	2,3E-02
*CACNA1S*	calcium channel, voltage-dependent, L type, alpha 1S subunit	2,5E-02	5,0E-02	3,4E-02
*CAMK4*	calcium/calmodulin-dependent protein kinase IV	6,3E-03	4,8E-02	4,6E-02
*CHRM2*	cholinergic receptor, muscarinic 2	2,0E-02	4,7E-02	2,3E-02
*CHRM3*	cholinergic receptor, muscarinic 3	1,9E-02	4,0E-02	4,1E-02
*EGFR*	epidermal growth factor receptor	5,0E-03	3,5E-02	3,7E-02
*ERBB4*	v-erb-a erythroblastic leukemia viral oncogene homolog 4 (avian)	2,3E-02	1,3E-02	1,7E-02
*GNA14*	guanine nucleotide binding protein (G protein), alpha 14	3,9E-02	4,9E-02	3,7E-02
*GNAL*	guanine nucleotide binding protein (G protein), alpha activating activity polypeptide, olfactory type	7,9E-04	3,5E-03	2,7E-02
*GRM1*	glutamate receptor, metabotropic 1	5,0E-02	3,7E-02	1,9E-02
*GRM5*	glutamate receptor, metabotropic 5	2,3E-02	4,7E-02	1,6E-02
*ITPR1*	inositol 1,4,5-trisphosphate receptor, type 1	3,5E-02	2,2E-02	2,1E-02
*ITPR2*	inositol 1,4,5-trisphosphate receptor, type 2	1,9E-02	4,2E-02	3,2E-02
*LHCGR*	luteinizing hormone/choriogonadotropin receptor	1,6E-02	3,7E-02	3,9E-02
*NOS1*	nitric oxide synthase 1 (neuronal)	3,0E-02	1,6E-02	4,7E-02
*P2RX7*	purinergic receptor P2X, ligand-gated ion channel, 7	4,7E-02	3,4E-02	4,0E-02
*PDE1A*	phosphodiesterase 1A, calmodulin-dependent	4,5E-02	2,7E-02	3,3E-02
*PDE1C*	phosphodiesterase 1C, calmodulin-dependent 70kDa	6,4E-03	4,2E-02	4,3E-02
*PLCB1*	phospholipase C, beta 1 (phosphoinositide-specific)	2,1E-03	4,6E-02	1,7E-02
*PLCB4*	phospholipase C, beta 4	4,4E-02	3,7E-02	1,3E-03
*PLCG2*	phospholipase C, gamma 2 (phosphatidylinositol-specific)	2,2E-02	1,2E-02	4,0E-02
*PLCZ1*	phospholipase C, zeta 1	4,5E-02	2,7E-02	3,7E-02
*PRKCA*	protein kinase C, alpha	3,6E-02	3,5E-02	1,4E-02
*PRKCB*	protein kinase C, beta	4,4E-02	2,2E-03	3,3E-02
*PTGER3*	prostaglandin E receptor 3 (subtype EP3)	3,3E-02	3,3E-02	3,6E-02
*RYR1*	ryanodine receptor 1 (skeletal)	4,7E-02	3,1E-02	1,8E-02
*RYR2*	ryanodine receptor 2 (cardiac)	3,9E-02	3,2E-02	1,6E-02
*RYR3*	ryanodine receptor 3	2,0E-02	2,9E-02	4,8E-02
*SLC8A1*	solute carrier family 8 (sodium/calcium exchanger), member 1	2,1E-02	4,5E-02	2,7E-02
*TACR1*	tachykinin receptor 1	5,4E-03	3,1E-02	1,5E-02
